# Interactions Between the Gut Microbiota and the Host Innate Immune Response Against Pathogens

**DOI:** 10.3389/fimmu.2019.00607

**Published:** 2019-03-29

**Authors:** Hong-Yu Cheng, Meng-Xia Ning, De-Kun Chen, Wen-Tao Ma

**Affiliations:** Veterinary Immunology Laboratory, College of Veterinary Medicine, Northwest Agriculture and Forestry University, Yangling, China

**Keywords:** gut microbiota, host innate immunity, antimicrobial peptides, inflammasome, IL-22, IL-17, IL-10

## Abstract

The mammalian intestine is colonized by over a trillion microbes that comprise the “gut microbiota,” a microbial community which has co-evolved with the host to form a mutually beneficial relationship. Accumulating evidence indicates that the gut microbiota participates in immune system maturation and also plays a central role in host defense against pathogens. Here we review some of the mechanisms employed by the gut microbiota to boost the innate immune response against pathogens present on epithelial mucosal surfaces. Antimicrobial peptide secretion, inflammasome activation and induction of host IL-22, IL-17, and IL-10 production are the most commonly observed strategies employed by the gut microbiota for host anti-pathogen defense. Taken together, the body of evidence suggests that the host gut microbiota can elicit innate immunity against pathogens.

## Introduction

The mammalian intestine is home to a complex and dynamic population of microorganisms, termed the “gut microbiota” (1, 2). These microorganisms, which co-evolved with the host as part of a mutually beneficial relationship (3), include bacteria, fungi and viruses (4, 5). Accumulating evidence indicates that the gut microbiota can participate in the maturation and function of the innate immune system, while also playing many complex roles in the host defense against pathogens (6). On the one hand, the gut microbiota can help repair intestinal mucosal barrier damage (7, 8); on the other hand, gut microbiota mediates host anti-pathogen defenses (9).

In the past decade, studies of germ-free (GF) mice have provided clues to elucidate the complexity of the intestinal microbiota (10, 11) and its importance to host health (12, 13). Mounting research shows that at least a thousand different gut microbiota species, such as *Firmicutes, Bacteroidetes, Actinobacteria, Proteobacteria*, and others, contribute to host defense against harmful microorganisms (14, 15).

Recently, several studies have begun to elucidate the molecular mechanisms underlying how the gut microbiota regulates host innate immunity against pathogens (16, 17), including a role in helping the host resist pathogen colonization. In this review, we summarize the main mechanisms by which commensal bacteria, including certain probiotic species, actively prevent pathogen colonization of the host.

## Gut Microbiota and Antimicrobial Peptides

### Defensins

The α-defensins, microbicidal peptides mainly produced by Paneth cells, are key components of innate immunity. They control pathogen growth within the intestine (18–20) and their production can be directly elicited by both Gram-negative and Gram-positive bacteria, as well as by bacterial metabolites (e.g., lipopolysaccharide, lipoteichoic acid, lipid A, and muramyl dipeptide) (21–23). By contrast, live fungi and protozoa do not appear to stimulate Paneth cells and thus fail to elicit Paneth cell degranulation (21). Nevertheless, recent research has found that the gut microbiota plays an important role in induction of α-defensins expression against pathogens (24). In one *in vitro* study, live *E. coli* or *S. aureus*, live or dead *S. typhimurium*, lipopolysaccharide (LPS), lipid A, lipoteichoic acid (LTA), or liposomes could stimulate isolated intact intestinal crypts, demonstrating that intestinal Paneth cells may contribute to α-defensins secretion by sensing the presence of exogenous bacteria and bacterial antigens (21). To investigate whether gut microbiota possess the same or similar functions, Shipra Vaishnava and colleagues used a CR2-MyD88 Tg mouse model, whereby Paneth cells were the sole cell lineage expressing MyD88, to demonstrate that Paneth cells may directly sense enteric bacteria to trigger the MyD88-dependent antimicrobial program. Furthermore, increased numbers of *Salmonella* were observed to be internalized by mesenteric lymph node (MLN) cells of MyD88^−/−^ and germ-free mice as compared to corresponding numbers observed for wild-type mice (25). Similarly, transcriptional profiles have shown that α-defensin gene (*Defa)* transcripts were less abundant in intestinal microbiota-free mice and TLRs-deficient or MyD88-deficient mice, but could be recovered after stimulation with toll-like receptor (TLR) agonists, specifically agonists of TLR2 or TLR4 (26). Thus, commensal microbiota appears to protect the host against pathogen invasion by triggering enteric Paneth cell TLR-MyD88 signaling. Notably, this mechanism is distinct from the NOD2-dependent antimicrobial response (25, 27, 28), since the former mechanism entails triggering of expression of multiple antimicrobial factors (25). However, several human-based studies have demonstrated that mutations in the NOD2 peptidoglycan sensor actually did reduce secretion of α-defensins (29–33). Therefore, these contradictory human and mouse study results warrant further research. Notably, another study has demonstrated that Cd1d^−/−^ mice exhibited a defect in Paneth cell granule ultrastructure that specifically resulted in an inability to degranulate after bacterial colonization, with an increased load of segmented filamentous bacteria (SFB) also noted (34). Thus, no clear evidence demonstrates that CD1d mediates regulation of gut microbiota via α-defensins expression.

Meanwhile, more recent research has begun to examine the mechanism of how the gut microbiota influences α-defensins secretion. Studies using the Caco-2 IEC line have demonstrated that lactic acid strongly suppresses transcription of the α-defensin gene, while cecal content may include as yet unidentified factors which enhance concomitant α-defensin 5 expression (35). However, contrary to the aforementioned results, Menendez et al. found that *Defa* expression was partially restored *in vivo* by *lactobacillus* administration to antibiotic-treated mice (26). Notably, an emerging role of vitamin D, a *lactobacillus* metabolite, has been recently discovered that exerts an effect opposite on α-defensins expression to that exerted by lactate (36, 37). To reconcile these results, Su et al. used a mouse model and certain feed formulations to demonstrate that VDD- and HFD ± VDD-fed mice exhibited reduced levels of expression of α-defensin and MMP7 (a metalloproteinase that can proteolytically convert pro-α-defensins to their mature and active forms) within ileal crypts as compared to results for control and HFD groups. Moreover, their results demonstrated a critical role of vitamin D signaling in maintaining steady-state expression of α-defensins and MMP7 under physiological conditions. Subsequently, Su et al. have demonstrated that dietary vitamin D deficiency resulted in loss of Paneth cell-specific α-defensins, which may lead to intestinal dysbiosis and endotoxemia (38). Of note, oral administration of α-defensin suppressed *Helicobacter hepaticus* growth *in vivo* (38). Meanwhile, using complementary mouse models of defensin deficiency (MMP7^−/−^) and surplus (HD5^+/+^), Salzman noted defensin-dependent reciprocal shifts in proportions of dominant bacterial species within the small intestine with no changes in total bacterial numbers observed (Table 1). Upon further research, this group observed that mice overexpressing HD5 exhibited a significant loss of segmented filamentous bacteria (SFB), resulting in reduced numbers of Th17 cells within the lamina propria (48). However, direct evidence implicating the involvement of SFB in α-defensin production is still lacking and studies on α-defensin regulation by specific commensal microorganisms are still rare, warranting further research. Nevertheless, in view of existing research results, we believe that the discovery of specific microorganisms through research focusing on specific metabolic pathways may be a more fruitful approach.

**Table 1:** Gut microbiota protects the host against pathogen infections and the relevant mechanisms.

**Pathogens**	**Gut microbiota**	**Mechanisms**	**References**
*Helicobacter hepaticus*	*Lactobacillus*	Inducing α-defensin production from Paneth cells	(38)
*S. aureus S. pyogenes P. aeruginosa E. coli C. albicans*	*Lactobacillus acidophilus PZ 1129 Lactobacillus acidophilus PZ 1130 Lactobacillus paracasei Lactobacillus plantarum E. coli K-12 E. coli Nissle 1917*	Inducing β-defensin production	(39–42)
*Klebsiella pneumoniae Citrobacter rodentium Enterococcus Plasmodium chabaudi*	*L. reuteri Allobaculum spp Clostridium spp Bacteroides spp*	Inducing IL-22 production	(43–46)
*Salmonella typhimurium*	*Bacteroides*	Inducing IL-17 production	(47)

With regard to β-defensins, which directly kill or inhibit the growth of microorganisms (49), these agents have been shown to exert antimicrobial activity against some species of enteric pathogenic Gram-positive *S. aureus* and *S. pyogenes*, as well as against Gram-negative *P. aeruginosa, E. coli* and the yeast *C. albicans* (50). In fact, accumulating evidence has shown that, similarly to α-defensins, β-defensins secretion is also regulated by the gut microbiota. For example, using *in vitro* studies of HT-29 and Caco-2 human colon epithelial cell lines, human fetal intestinal xenografts have been observed to constitutively express hBD-1 but not hBD-2, with upregulation of only the latter in xenografts intraluminally infected with *Salmonella* (51). Meanwhile, it has been independently shown that preincubation of Caco-2 cells with live *E. faecium* significantly reduced *S. typhimurium* internalization by 45.8%, while heat-killed *E. faecium* pretreatment had no effect on pathogen internalization (49). This result aligns with the latest research, which has shown that only live gut microbiota, as modeled using *Lactobacillus acidophilus* PZ 1129 and PZ 1130, *Lactobacillus paracasei, Lactobacillus plantarum, E. coli* K-12, and *E. coli Nissle 1917*, can strongly induce expression of hBD-2 in Caco-2 intestinal epithelial cells in a time- and dose-dependent manner (39–42) (Table 1). Notably, the *E. coli* strain *Nissle 1917*, a non-pathogenic Gram-negative strain isolated in 1917 by Alfred Nissle, elicited the most marked expression of induced β-defensin expression *in vitro* (39–42). Interestingly, Schlee et al. constructed several *E. coli Nissle 1917* deletion mutants and pinpointed flagellin as the major stimulatory factor for triggering of β-defensin secretion in the presence of that strain (40). Meanwhile, Wehkamp et al. and others have found that *E. coli Nissle 1917*-induced β-defensin expression in cell culture was mediated by NF-κB- and MAPK/AP-1-dependent pathways (39–42). Nevertheless, *in vivo* studies are still needed to confirm if gut microbiota can induce β-defensins expression to reduce pathogen colonization and control gut homeostasis (Table 1). Recently, to further clarify the relationship between gut microbiota and β-defensin secretion, Miani et al. used a mouse model and antibiotic treatment experiments to study the participation of dysbiotic microbiota and a low-affinity aryl hydrocarbon receptor (AHR) allele in the defective pancreatic expression of mBD14 observed in NOD mice. By utilizing 16S rDNA gene sequencing and AHR ligand activity measurements, they demonstrated that gut microbiota-derived molecules, including AHR ligands and butyrate, promoted IL-22 secretion by pancreatic ILCs that subsequently induced mBD14 expression by endocrine cells. Therefore, dysbiotic microbiota and a low-affinity AHR allele appear to explain defective pancreatic mBD14 expression of mBD14 in NOD mice (24). Because only live gut microbiota can stimulate secretion of β-defensins, we believe that specific gut microbiota that possess special metabolic pathway functionality, including pathways for secretion of AHR ligands, may possess the ability to regulate secretion of β-defensins.

### C-Type Lectins

**Figure 1:**
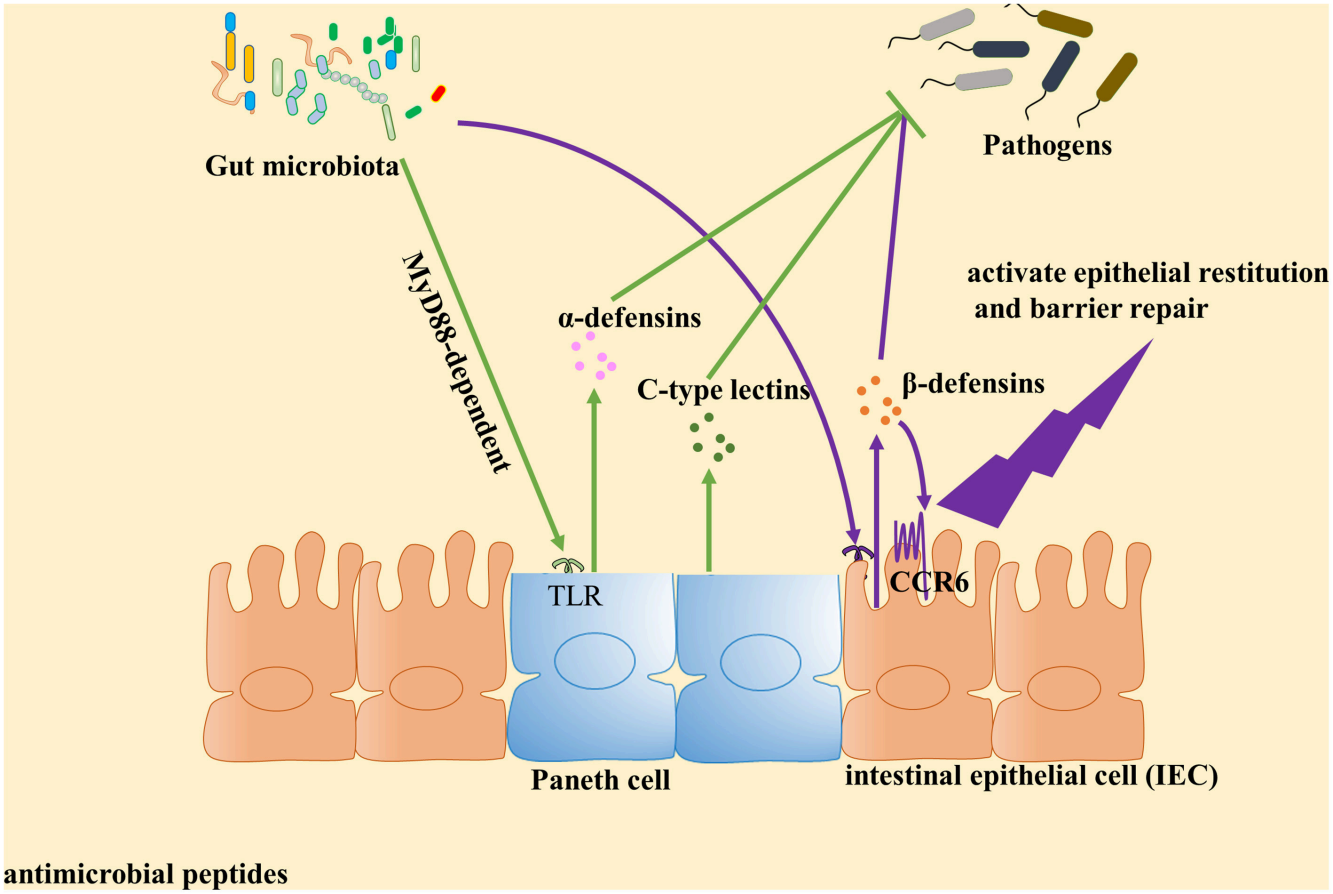
Gut microbiota plays an important role in the induction of antimicrobial peptides expression against pathogens. Antimicrobial peptides are key components of innate immunity to control pathogen growth within the intestine. Accumulated evidence identified that gut microbiota can contribute to the expression of antimicrobial peptides and play a central role in host defense against pathogens. Paneth cells could directly sense gut microbiota through cell-autonomous myeloid differentiation primary response 88 (MyD88)-dependent toll-like receptor (TLR) activation, triggering expression of α-defensins and C-type lectins. With regard to β-defensin, gut microbiota induce β-defensin expression in cell culture mediated by NF-κB- and MAPK/AP-1-dependent pathways *in vitro*. Then β-defensin interacts with intestinal epithelial cell (IEC) through CCR6 to activate epithelial restitution and barrier repair.

The C-type lectins, also key components of innate immunity that control growth of enteric pathogens (52–54), are expressed by multiple small intestinal epithelial lineages (55, 56). REG3γ and REG3β, two C-type lectins, provide protection against infection by specific bacterial pathogens, including *Enterococcus faecalis* (57–59), *Yersinia pseudotuberculosis* (60, 61), and *Listeria monocytogenes* (57). Notably, additional evidence suggests that C-type lectins actually mediate syncytium endosymbiont defenses through prevention of pathogen colonization. To further demonstrate how these lectins control bacterial colonization of the intestinal epithelial surface, Vil-Myd88^Tg^ mice (mice with IEC-restricted Myd88 expression) were used to determine whether surface Myd88 present on epithelial cells was sufficient to restrain bacterial colonization (55). The results showed that secretion of C-type lectins required both activation of the MyD88 pathway (62) and recognition of syncytium endosymbionts by TLRs (63). Furthermore, Earle et al. used a pipeline method to assess intestinal microbiota localization within immunofluorescence images of fixed gut cross-sections. The results indicated that elimination of dietary microbiota-accessible carbohydrates (MACs) resulted in thinning of mucus within the distal colon that increased microbial proximity to the epithelium and heightened inflammatory marker REG3β expression (64). These results align with those from an earlier study of transcriptional profiles of duodenum, jejunum, ileum and colon samples, which demonstrated that MyD88 was essential for syncytium endosymbiont-induced colonic epithelial expression of antimicrobial genes Reg3β and Reg3γ, with Myd88 deficiency associated with both a shift in bacterial diversity and a greater proportion of SFB in the small intestine (65). In fact, other research found that conventionally raised Myd88^−/−^ mice exhibited increased expression of antiviral genes in the colon, which correlated with norovirus infection of the colonic epithelium (65). Therefore, it can be concluded that both the activation of the MyD88 pathway and recognition of syncytium endosymbionts by TLRs are indispensable for triggering C-type lectins secretion (Figure 1). Recently, Ju et al. used antibiotic-treated mice to study differences between metronidazole-treated and control groups, and observed reduced abundance of *Turicibacteraceae*, overgrowth of *E. coli* and higher levels of *Reg3*β and *Reg3*γ mRNA for the metronidazole-treated group (66). These results provide a basis for the study of the effects of specific gut syncytium endosymbiont organisms on C-type lectins secretion.

Other accumulating evidence has shown that the mammalian gut contains a rich fungal community that interacts with the immune system through the C-type lectin receptor Dectin-1. To demonstrate whether symbiotic fungi influence C-type lectins secretion that prevents pathogen colonization, Iliev et al. studied mice lacking Dectin-1 and observed increased susceptibility to chemically induced colitis due to altered responses to indigenous fungi. Moreover, in humans they identified a gene polymorphism for Dectin-1 (CLEC7A) that is strongly linked to a severe form of ulcerative colitis (67). Independently, Eriksson et al. found that CLR-specific intracellular adhesion molecule-3 grabbing non-integrin homolog-related 3 (SIGNR3) is the closest murine homolog to the human dendritic cell-specific intercellular adhesion molecule-3-grabbing non-integrin (DC-SIGN) receptor. Both receptors recognize similar carbohydrate ligands, such as terminal fucose or high-mannose glycans. Notably, using the dextran sulfate sodium-induced colitis model, IGNR3 has been observed to recognize fungal members of the commensal microbiota, with SIGNR3^−/−^ mice exhibiting a higher level of TNF-α in colon (68). Therefore, symbiotic fungi appear to communicate with the host via the C-type lectin receptor to maintain intestinal homeostasis. However, as yet no direct evidence has been found to determine whether symbiotic fungi can regulate selectin secretion, warranting further research.

## Gut Microbiota Elicits Inflammasome Activation Against Pathogens

Inflammasome activation is an important innate immune pathway that prevents pathogen invasion via secretion of proinflammatory cytokines IL-1β and IL-18, as well as through induction of pyroptosis (69–74). It is well-documented that inflammasomes come from two main sources, namely myeloid- and epithelial-derived inflammasomes. While they share several common features, it should be noted that inflammasomes of distinct origins may exhibit different features and effector functions. For example, from a mechanistic of view, macrophage- and epithelial cell-derived inflammasomes are activated with different intermediate processes. While IL-18 processing is dependent on caspase-11 in IECs, caspase-1 is responsible for the processing of IL-18 in myeloid cells (75). In addition, compared with myeloid cells, IECs constitutively express IL-18, while produce little IL-1β (76–78). Moreover, unlike myeloid inflammasomes, IEC inflammasomes is capable of producing considerable amounts of prostaglandin upon activation (79). Intriguingly, the signaling circuitry between epithelial and myeloid inflammasomes are also different. For example, in homeostasis conditions, both *NLRP3* and *PYCARD* genes have been shown to be highly expressed in murine primary macrophages, while mouse airway epithelial cells can only express a low level of *PYCARD* and cannot express *NLRP3* (80).

Accumulating evidence suggests that gut microbiota can activate NLRC4 and NLRP3 inflammasome pathways against pathogens (81–83). *Enterobacteriaceae* and the pathobiont *Proteus mirabilis*, which are members of the normal flora of the human gastrointestinal tract (84, 85), were shown to induce robust IL-1β production through NLRP3 activation mediated by intestinal Ly6C^high^ monocytes (86, 87). Indeed, recruited Ly6C^high^ monocytes have been shown to express a variety of inflammasome components, such as NAIPs (71, 88, 89), NLRC4 (89), NLRP1 (90, 91), NLRP6 (92, 93), AIM2 (94), caspase-1 (95), caspase-4 (96) (in humans), ASC (93), and IL-18 (87, 97, 98). Meanwhile, Seo et al. have also demonstrated that *Proteus mirabilis* (a Proteobacteria phylum member) induced NLRP3 activation and IL-1β production (86). Interestingly, bacterial components from other *Proteobacteria*, such as LPS produced by *Pseudomonas* spp., have even been shown to induce host mental depression symptoms via NLRP3 inflammasome activation (99). Other interesting lines of research have shown that in addition to gut commensal bacteria, the mammalian gut contains a rich fungal community which also appears to activate the inflammasome pathway. This community includes the human commensal fungus *Candida albicans* (*C. albicans*), which colonizes gastrointestinal and vaginal tract mucosal surfaces and appears to promote inflammasome activation during AOM-DSS-induced colitis (100). In further support of this finding, direct peptide administration experiments had previously demonstrated that candidalysin, a peptide derived from the hypha-specific *ECE1* gene, acted as a fungal trigger for NLRP3 inflammasome-mediated maturation that was sufficient for inducing IL-1β secretion mature macrophages in an NLRP3 inflammasome-dependent manner (101).

In recent studies, numerous other gut microbiota metabolites have also been demonstrated to elicit inflammasome pathways against pathogens. For example, gut microbiota-derived adenosine triphosphate (ATP) has been shown to co-operate with NLRP3 (also known as CIAS1) (102) via the macrophage P2X7 receptor (103) to induce assembly of a cytosolic protein complex containing ASK and caspase-1 (70, 104–106) that eventually leads to inflammasome activation (106). Another important gut microbiota metabolite, short-chain fatty acids (SCFAs), end products of fermentation of dietary fibers by anaerobic intestinal microbiota, have also been implicated in inflammasome activation (107). SCFAs binding to GPR43 on colonic epithelial cells to stimulate K^+^ efflux and hyperpolarization has been shown to lead to NLRP3 inflammasome activation, with subsequent acceleration of cell maturation and secretion of IL-1β (108) and IL-18 (77, 109).

## Gut Microbiota Can Enhance Interleukin Expression to Clear Invading Pathogens

### IL-22

**Figure 2:**
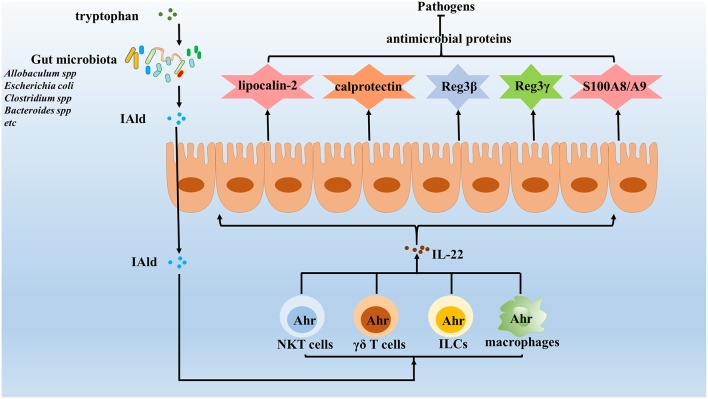
Gut microbiota enhance the expression of IL-22 against invading pathogens. IL-22 is important in maintaining the integrity of mucosal barriers and can be produced by many different types innate immune cells, which induce the expression of various antimicrobial proteins, including lipocalin-2, calprotectin, C-type lectins, S100A8, S100A9, and so on to clear pathogens. Increasing evidence identified that gut microbiota enhances the expression of IL-22 to protect the host against pathogens. The results show that gut microbiota utilize tryptophan as an energy source and produce a metabolite, indole-3-aldehyde (IAld), which in turns activated Ahr on innate immune cells. Once activated, innate immune cells will secrete IL-22, which protects the host against opportunistic pathogens by reducing their colonization.

IL-22 is important in maintaining mucosal barrier integrity and is produced by many different types of innate immune cells (110–113). This cytokine has been shown to play a host-protective role during infection by a wide range of pathogens, including *Klebsiella pneumoniae* (114), *Citrobacter rodentium* (115, 116), vancomycin-resistant *Enterococcus* (117, 118) and *Plasmodium chabaudi* (119). One IL-22-dependent mechanism involved in pathogen clearance involves the increased presence of antimicrobial proteins within the mucosa (120) that include the following: calprotectin and lipocalin-2, the latter of which binds to the siderophore enterochelin, with both acting to limit iron availability in the gut (120); C-type lectins, which regenerate islet-derivative proteins Reg3β and Reg3γ that control some components of the microbiota (58, 120, 121); and S100A8 and S100A9, two antimicrobial peptides that heterodimerize to form calprotectin, an antimicrobial protein that sequesters zinc and manganese to prevent microbial access to these nutrients (122). Although epithelial antimicrobial defenses also exist, many pathogens can still colonize mucosal surfaces to establish infections (120, 123). Nevertheless, accumulated evidence has shown that IL-22 is rapidly induced in response to pathogen invasion through activation of host AhR via specific gut microbiota-derived molecules (Figure 2) (124, 125). For example, *Lactobacillus species* (specifically, *L. reuteri*) can activate IL-22 production by gut type 3 innate lymphoid cells (ILC3) (126–128), while other studies have shown that supplementation with three commensal *Lactobacillus strains* with high tryptophan-metabolizing activities was sufficient to restore intestinal IL-22 production (43, 129). Indeed, additional work has shown that *Lactobacillus species* could utilize tryptophan as an energy source and produce a metabolite, indole-3-aldehyde (IAld), which could then activate AhRs present on ILCs (126, 130). In addition to *Lactobacillus strains*, other recent studies have shown that *Allobaculum* spp. (43), *Escherichia coli* (44)*, Clostridium* spp. (45), and *Bacteroides* spp. (46) can also utilize tryptophan to produce IAld and elicit IL-22 production (Table 1). Meanwhile, other studies have shown that activated ILCs secrete IL-22 to protect the host against opportunistic pathogens by reducing pathogen colonization (120, 131). In fact, other innate immune cells, such as NKT cells, γδ T cells and macrophages, have very recently been shown to secrete IL-22 under regulation by gut microbiota via the AhR pathway (132). Therefore, gut microbiota may prevent pathogen infection by collectively enhancing IL-22 expression via the AhR pathway.

### IL-17

IL-17 is a well-established crucial cytokine that is involved in limiting invasion and dissemination of pathogens, including *Salmonella typhimurium* (133), by both recruitment of neutrophils and by the induction of production of antimicrobial peptides (131, 134). Recent studies have demonstrated that both the abundance and activation status of IL-17-producing intraepithelial lymphocytes (IELs) are modulated by commensal bacteria, with enrichment of the γδT cell population of IELs representing an important source of innate IL-17 production (135, 136). Notably, a comparative study of GF mice and SPF mice has shown that the number of TCRγδ IELs is decreased in GF mice (133). Moreover, in addition to the regulation of IELs numbers, the gut microbiota may also regulate activation of TCRγδ IELs, as reflected by a report showing that production of IL-17 by TCRγδ IELs is decreased in GF mice (137). Meanwhile, antibiotic-treatment and monocolonization of mice have been used to demonstrate that the great majority of γ/δ T cells within peritonea of SPF mice are CD62L^−^ γδT cells, which are activated γδT cells, with GF mice possessing far fewer CD62L^−^ γ/δ T cells than SPF mice (47). Notably, additional research suggests that specific commensal bacteria, excluding metronidazole-sensitive anaerobes, such as *Bacteroides* species, are required for maintaining IL-1R1^±^ γδT cells (47), a result that aligns with previous research results by another research group (138) (Table 1). In conclusion, gut microbiota influences the abundance and activation status of IL-17-producing TCRγδ IELs to protect the host from pathogen infection and to maintain intestinal homeostasis. In addition, lymphoid tissue inducer (LTi) cells and NCR^−^ ILC3 cells also appear to function as important sources of innate IL-17 production (127). However, few studies have investigated how gut microbiota regulate these cell types, warranting further research in this area.

### IL-10

IL-10 is an anti-inflammatory cytokine that plays a central role in regulating the host immune response to pathogens, thereby preventing host damage and maintaining normal tissue homeostasis (139–141). Accumulating evidence suggests that macrophages are an important source of innate IL-10 and that the gut microbiota plays a vital role in mucosal innate IL-10 generation under homeostatic conditions (142–144). For example, studies in GF mice and SPF mice have shown that colonic lamina propria from germ-free mice exhibited lower IL-10 production (142), a reduction later confirmed to be a 50% reduction in steady-state IL-10 levels (142–144). To elucidate the mechanism by which gut microbiota regulate intestinal macrophage IL-10 production, Hayashi et al. used macrophage-specific IL-10-deficient mice to demonstrate that *Clostridium butyricum* (CB), a distinct cluster I *Clostridium* strain, induces IL-10 production to ultimately prevent acute experimental colitis. However, while CB treatment had no effects on IL-10 production by T cells, IL-10-producing F4/80^±^CD11b^±^CD11c^int^ macrophages accumulated within inflamed mucosa after CB treatment. Subsequently, more rigorous examination demonstrated that CB directly triggered IL-10 production by intestinal macrophages there via the TLR2/MyD88 pathway (144). Meanwhile, Ochi et al. recently found that dietary amino acids directly regulate Il-10 production by small intestine (SI) macrophages. Using mice fed via total parenteral nutrition, a significant decrease of IL-10-producing macrophages in the SI was observed, while IL-10-producing CD4^±^ T cells remained intact. Likewise, enteral nutrient deprivation selectively decreased IL-10 production by the monocyte-derived F4/80^±^ macrophage population, but had no effect on non-monocytic precursor-derived CD103^±^ dendritic cells. Notably, in contrast to regulation of colonic macrophages, replenishment of SI macrophages and their IL-10 production were not regulated by gut microbiota (145). Contrary to results obtained under steady-state conditions, an injury model used to study participation of microbiota to explain observed IL-10 increases post-injury yielded different results. Specifically, comparison of *Il10* mRNA levels in uninjured intact tissue and day-2 post-wound tissue isolated from SPF or GF mice indicated that IL-10 mRNA was induced in post-wound colonic tissue isolated from both SPF and GF mice. Therefore, injury-triggered IL-10 increases appeared to be largely microbiota independent (146), although the reasons remain unclear regarding the differing effects of the gut microbiota observed in different model systems. Nevertheless, we hypothesize that local damage-associated molecular proteins (DAMPs) may regulate immune cells more rapidly and strongly post-intestinal damage, resulting in either a failure of gut microbiota to temporally adjust or a masking of any microbiota-based regulatory effect.

## Concluding Remarks

Gut microbiota resists colonization and growth of invading pathogens through the induction of expression of antimicrobial peptides, IL-22, IL-17, and IL-10 while eliciting inflammasome activation. Because the underlying mechanisms of how the gut microbiota resists pathogenic invasion still remain obscure, future studies are clearly needed to identify gut microbiota functions against various pathogens toward the development of promising strategies to treat infectious diseases. For instance, E. coli Nissle 1917 can induce β-defensin expression mediated by NF-κB- and MAPK/AP-1-dependent pathways (39), while Lactobacillus spp. activate IL-22 production against opportunistic pathogens to reduce colonization (147, 148). Therefore, transplanting suitable specific gut microbiota to compete with specific pathogens could be an effective defense strategy. However, since this strategy poses new disease risks, strategies that restore intestinal homeostasis and promote host immune system may serve to more safely clear pathogens. To this end, identifying specific gut microbiota functions and defining normal gut microbiota populations are necessary first steps toward development of safer strategies for strengthening host defenses against pathogens. Moreover, research on the function and mechanisms of gut microbiota metabolites may facilitate development of novel therapeutic strategies to combat drug-resistant pathogens.

## Author Contributions

D-KC, W-TM, H-YC, and M-XN designed the structure of the mini-review. H-YC and M-XN wrote the manuscript and drafted the first version of the manuscript. M-XN and W-TM helped revise the manuscript. All authors have reviewed the final version of the manuscript.

### Conflict of Interest Statement

The authors declare that the research was conducted in the absence of any commercial or financial relationships that could be construed as a potential conflict of interest.
